# Affective integration in climate change communication: recency effects and negativity bias in responses to climate picture sequences

**DOI:** 10.3389/fpsyg.2026.1797253

**Published:** 2026-09-21

**Authors:** Hulda Karlsson-Larsson, Erkin Asutay, Daniel Västfjäll

**Affiliations:** 1 Department of behavioural sciences and learning, JEDI-lab, Linköping University, Linköping, OR, Sweden; 2 Decision Research Eugene, OR, United States

**Keywords:** affect, affective integration, climate change pictures, emotion, pro-environmental behavior

## Abstract

This research explored climate pictures as affect-eliciting stimuli, and how the affective experiences can have an impact on pro-environmental intentions. In an online within-subject design experiment (*N* = 464), participants engaged in twelve experimental trials, each containing one picture of a wildfire, one of a forest, and one depicting a pro-environmental action, presented in various orders. Following each trial, participants rated their affective experiences and pro-environmental intentions. The results indicated that affective valence (positive-negative experience) was sensitive to the presentation order of the pictures. Using a quasi-Bayesian approach to mediation analysis, we found that the presentation order indirectly influenced pro-environmental intentions through changes in affective valence. Furthermore, hierarchical Bayesian modeling revealed that normative valence ratings of pictures, picture type, and the position of the pictures in the presentation order (first, second, or third) predicted the affective valence reported by participants. Specifically, the model highlighted a significant recency effect, indicating that the final picture in each sequence exerted a greater impact on the reported affective valence. Additionally, while all picture types predicted affective valence, wildfire pictures exerted a stronger influence on affective experience than forest pictures, consistent with negativity bias, proposing that negative information carries greater weight than positive information. These findings suggest that affective responses to climate change communication are shaped by both recency effects and the stronger weighting of negative relative to positive climate-related stimuli, highlighting the importance of understanding how multiple affective inputs jointly influence behavioral motivation. These results suggest that our computational model of affect can inform the design of climate change communication strategies to more effectively leverage emotional responses to motivate pro-environmental action.

## 1 Introduction

A growing body of research underscores the pivotal role of affect and emotion in addressing climate change, particularly in motivating pro-environmental action (Asutay et al., 2023; Brosch, 2021; Brosch and Sauter, 2023; Goldberg, 2023; Karlsson et al., 2023; Schneider et al., 2021; Van Der Linden, 2018). Emotion can influence decision making and behavior (Bechara et al., 2000; Slovic and Peters, 2006; Slovic et al., 2007). Furthermore, it has been proposed that emotion functions as a mechanism in reinforcement processes, whereby emotional experiences influence future behavior (Baumeister et al., 2007; Bechara et al., 2000; Berridge, 2012). Building on the concept of emotion as an indirect (i.e., *via* antecedents) motivator for behavior (Baumeister et al., 2007; Brosch, 2021; Schneider and van der Linden, 2023), this study explored how affective responses to affect-eliciting stimuli (climate change pictures) impact pro-environmental intentions. Specifically, the present study focused on core affect, a constantly fluctuating state of valence and arousal that forms a fundamental component of emotional experience (Barrett, 2017), because it allows for the continuous assessment of affective responses across picture sequences.

Affective experience is a dynamic process where, often, a stream of different internal and external inputs is summarized into an overall experienced state. This state in turn motivates and demotivates behavior (Asutay and Västfjäll, 2024). The present research therefore focuses on the affective experiences elicited by a sequence of pictures relating to different sources of affective input related to climate change (pro-environmental actions, climate change consequences, and positive environmental outcomes). By manipulating the affective valence and type of these pictures, we evaluated how different sequences influence key psychological antecedents of pro-environmental behavior, including affect, impact beliefs (i.e., beliefs about mitigation potential), perceived climate change risk, perceived behavioral control, and pro-environmental intentions.

### 1.1 The role of climate change pictures in eliciting affect

Climate change pictures are one type of affect-eliciting stimuli that can be used to understand the relationship between affect and pro-environmental action. To explore the potential of pictures in promoting behavioral change, it is crucial to understand what affective reactions are elicited by climate change related pictures and how these reactions help motivate behavior. Climate change pictures, such as pictures of polar bears on melting ice sheets, are frequently used in communication efforts because they elicit stronger affective experiences than text-based content, thereby enhancing their effectiveness in campaigns (Casas and Williams, 2019). To increase the understanding of the effect of these types of stimuli, a stimulus bank with normed climate change pictures was created, where each picture was rated on valence, arousal, and climate change relevance (Lehman et al., 2019). Lehman et al. (2019) found that the pictures rated as more relevant to climate change elicited more negative valence and higher arousal. Additional research on climate change pictures has found that pictures showing climate change consequences (i.e., floods) produced negative affective reactions, while action pictures (i.e., using solar energy) elicited positive responses (Chapman et al., 2016). These studies demonstrate that the core affective experience can be manipulated through climate change pictures.

Beyond manipulating affect, knowledge about the subsequent effect of climate change pictures on other psychological antecedents for behavior is needed. (O'Neill and Hulme 2009) compared expert-generated pictures (e.g., the melting of the West Antarctic Ice Sheet) with non-expert pictures (e.g., polar bears), revealing that while expert pictures often led to feelings of disengagement, non-expert pictures fostered greater affective and cognitive engagement with climate change issues. Building on this, (O'Neill et al. 2013) later explored the difference between pictures showing the consequences of climate change (i.e., ice melting) and climate action (i.e., future energy solutions). Their study revealed that pictures of climate consequences increased perceived risk but decreased self-efficacy. Whereas action pictures increased both the reported risk and perceived efficacy. Somewhat inversely, Chapman et al. (2016) found that pictures of climate consequences (i.e., floods) increased the intention to act pro-environmentally, although efficacy and intention—is of course not the same construct.

Evidence from an attentional (dot-probe) task suggested that showing pictures of climate change action (e.g., solutions like solar panels) elicited positive affective valence, as well as effectively captured the attention of viewers (Carlson et al., 2020). They categorized this phenomenon as a type of attentional bias favoring positive pictures. Furthermore, the effect of this bias was found to be influenced by the degree of environmentalist identification, with individuals identifying more strongly as environmentalists exhibiting a reduced attentional bias. Interestingly, negatively valenced pictures—industrial pollution or natural disasters—did not capture people's attention. Instead, they were associated with a form of paralysis, consistent with the phenomenon of “freeze reactions” (i.e., slowed reaction times). Conversely, other evidence suggests that negatively valenced pictures (i.e., water pollution) are more attention–evoking (Salazar et al., 2022).

These findings highlight the importance of selecting appropriate pictures in climate change communication and underscore the complexity of the affective responses they can evoke. In addition to choosing which pictures to use, we argue that communicators must also consider the timing and target audience of their messages. However, previous research has typically studied the impact of climate change pictures in isolation.

In contrast, the current study explored how a sequence of photographic pictures influences psychological factors that lead to pro-environmental behavior. This approach suggests, as Markowitz and Guckian (2018) also note, that effective communication should be viewed as a process unfolding over time, creating an ongoing experience. Moreover, the affective integration of these pictures over time is not simply the sum of individual reactions (Asutay et al., 2020; Västfjäll et al., 2016), emphasizing the need for studies that explore climate change communication in a temporal context.

### 1.2 Affective integration

Affect functions as an internal feedback system, providing moment-to-moment updates on our state. The experiences of an individual's external and internal environment form an affective temporal sequence. However, research has consistently shown that not all moments in this sequence are attributed equal importance. In a seminal study, Kahneman et al. (1993) subjected participants to physical pain through cold water immersion. They found that people preferred to endure a longer period of unpleasantness if the end of the experience was less painful. From this, they identified the phenomenon of *duration neglect*—the tendency for individuals to place less weight on the total experience (i.e., affective sum) and instead focus on the most intense moment (i.e., peak effect) and the final part of the experience (i.e., end effect).

The end (or recency) and peak effects, have been replicated in a range of studies, more recently, using affective pictures (Asutay et al., 2020, 2021), smells (Scheibehenne and Coppin, 2020) and demanding working memory tasks (Hsu et al., 2018). Indeed, meta-analytic evidence find a strong stable effect (Alaybek et al., 2022). Further, recent evidence suggest that both effects seem to be modulated by sequence duration, in that they become more intense if the affective sequence is longer (Asutay et al., 2021; Li and Lapate, 2024). Memory-based accounts suggest that recently presented stimuli exert greater influence because they remain more accessible in memory during affective integration (Davelaar et al., 2005). Using a similar type of affect eliciting stimuli as the current study (i.e., manipulating valence with pictures), Asutay et al. (2020) showed that a simple averaging of the sequence of affective experiences fall short in predicting experienced affect. Instead, an average based model that allowed for higher weight to be put on the affective experiences associated with both peak and end moments were the most predictive model.

Beyond recency effects, negative information may exert a disproportionate influence on evaluation and behavior. According to the negativity bias, negative events tend to carry greater psychological weight than equally intense positive events, thereby exerting a stronger influence on attention, judgments, and decisions (Baumeister et al., 2001; Rozin and Royzman, 2001). Recent evidence from environmental psychology further indicates that emotions associated with environmentally harmful behaviors have a stronger influence on attitudes and behavioral decisions than emotions associated with environmentally friendly actions (Kesenheimer et al., 2026). In the context of affective integration, this suggests that the overall affective experience may depend not only on when affective information is encountered in a sequence, but also on the relative weight assigned to negative and positive affective inputs.

### 1.3 Present study

Previous literature has examined climate change consequence images, such as wildfires (Chapman et al., 2016; Lehman et al., 2019; O'Neill et al., 2013), and climate action in the form of solutions (Carlson et al., 2020; O'Neill et al., 2013). These studies have consistently shown that consequence images influence affective experience. Lehman et al. found that images of wildfires and industrial smog were rated as highly relevant to climate change and elicited strong negative valence and high arousal. Chapman et al. demonstrated that consequence images increased motivation to act, while O'Neill et al. showed that they increased perceived risk but reduced self-efficacy. Carlson et al. found that consequence images did not capture attention, but instead slowed reaction times.

Solution images, such as solar panels and windmills, have been shown to evoke positive affect and capture attention (Carlson et al., 2020), and to increase perceived self-efficacy (O'Neill et al., 2013). However, Chapman et al. found that these images were less motivating than consequence images. Importantly, solution images in previous studies have typically represented systemic or infrastructural change, not individual-level behaviors. Potentially allowing for a diffusion of responsibility (Gifford, 2011).

Goal images, such as healthy forests, have received less attention in climate change research. However, studies on mental health suggest that natural environments promote positive emotional responses (Dal Fabbro et al., 2021). These can represent a desirable future—a goal, but their role in shaping climate-related affect and behavior remains underexplored.

Recent research suggests that effective climate change communication often involves storytelling and clear steps to action (O'Callaghan et al., 2025). The use of image sequences in this study reflects how visual messages are typically encountered, in a temporal sequence. Our image selection built on findings that combining consequences, goals, and actions through mental imagery can strengthen intention (Karlsson et al., 2024). Including individual-level action images also drew on implementation intention research, which highlights the importance of linking intentions to specific, actionable cues (Armitage, 2004).

To date, no study has examined how these distinct image types—consequence, goal, and individual action—when presented in a sequence, and how these sequences influence affective experience and behavioral intention. The current study addressed this gap, in this within-subject online experiment, participants viewed three pictures per sequence—a wildfire (consequence), a healthy forest (goal), and a pro-environmental behavior (action). The serial order of the picture types varied across trials (first, second, and third). This design allowed us to test the idea that the integrated affective experience is more than the mere sum of individual moments. Specifically, we examined whether affective experience is shaped by both the temporal position of pictures within a sequence (i.e., recency effects) and differences in the affective weight assigned to negative and positive climate-related stimuli.

We hypothesized that each sequence of the presented climate change pictures would lead to different affective responses. In addition, we hypothesized the order in which pictures were presented would have a direct effect on pro-environmental intentions, as well as an indirect effect mediated by the unique affective experience elicited by each order. Lastly, we aimed to build a computational model that could predict participants' affective responses after viewing each picture sequence. Building on previous affect integration work (Asutay and Västfjäll, 2024), we hypothesized that affect could be predicted by prior affective experiences, normative affective ratings of the pictures, the order of presentation, and the type of behavior represented.

## 2 Method

### 2.1 Participants

We pre-registered (see OSF) to collect a minimum of 499 participants, based on power calculations for the longitudinal mediation analysis (high ICC, small effect size), which indicated that this sample size would provide 80% power for the analyses split by behavior (i.e., three observations per behavior; Pan et al., 2018). Although the recruitment target was 499 participants, data from 524 participants was collected before recruitment was closed.

The participants were recruited online (Prolific) from an American sample and responded through an online survey tool (Qualtrics). All data were collected during a single session (6–7th February 2024). To be included, participants had to be at least 18 years of age and have an approval rate of 95 % on the platform. Participants received monetary compensation for their participation. Informed consent was collected from all participants. According to guidelines from the Swedish Research Council concerning the Ethical Review of Research Involving Humans (SFS 2003:460), approval from an ethics committee is not required for behavioral research such as the study included in the manuscript.

Data from a total of 524 participants completed the full survey. After exclusions, the final sample used in the analyses consisted of 464 participants (88.55 % of the sample recruited). To a large part 57 participants were excluded due to viewing pictures longer than programmed in the experiment, we believe that this occurred due to insufficient internet connection. Each image was presented on a separate Qualtrics page with a six-second timer. Due to page load and submission delays, some recorded durations extended up to 12 s. To account for this, we allowed a two-second buffer (1 s for loading, 1 s for submission) and excluded participants who viewed any image for more than 8 s. We do not believe that viewing an image for up to two additional seconds would have meaningfully influenced participants' affective experience (Asutay et al., 2021). Some of the participants were excluded due to failing the attention check (*N* = 3). The final sample (*N* = 464) had the following gender distribution: females (39.66 %), males (58.19 %), non–binary/third gender (1.94 %), prefer not to say (0.22 %). The mean age in the final sample was 42.21 (*SD* = 13.38).

### 2.2 Procedure and materials

The experiment was a within-subject design, starting with a baseline rating followed by 12 trials. The baseline rating included measures of pro-environmental intention (four items, α = 0.66) and impact beliefs (four items, α = 0.87) related to four pro-environmental behaviors (avoid plastic, recycling, shift to public transport, and renewable electricity). Pro-environmental intention was measured using self-report items, where participants answered: “How willing are you to adopt these sustainable behaviors?”, on a scale from 0 (Not at all willing) to 100 (Extremely willing). To measure impact-belief participant responded to the question: “Do you believe that this sustainable behavior can have an impact on individual carbon emissions?”, on a scale from 0 (No impact) to 100 (Very high impact).

Following these ratings, participants answered questions regarding their perceived risk of climate change and their current affective experience measured using both valence and arousal. The perceived risk of climate change was measured with six items (α = 0.97) at baseline. Specifically, the measure included items related to perceived risk of climate change posed to oneself (two items), to others (two items), and non–human populations on earth (two items), all items were on a visual analogs scale (VAS scale) ranging from 0 (Climate change does not present any risk) to 100 (Climate change presents an extreme risk). Valence and arousal were measured using single items assessing the participants' level of pleasantness and activation respectively. Participants first read a text explaining both the valence scale and arousal scale and then answered the question, “How do you feel right now”. When rating valence, participants answered on a VAS scale ranging from 0 (Negative) to 100 (Positive), for arousal they answered on a VAS scale ranging from 0 (Low activation) to 100 (High activation). An attention check was included among the baseline measures.

#### 2.2.1 Experimental trials

ll trials included three climate change pictures, each presented for 6 s. The six-second presentation time was selected to provide participants with sufficient time to view and process each image while maintaining a continuous flow of affective inputs across the sequence. Each trial consisted of one photo of a healthy forest—labeled goal pictures, one of a wildfire—labeled consequence pictures, and one climate change action photo (Avoid plastic, Recycling, Shift to public transport, Renewable electricity) —simply labeled action pictures. The presentation order of the three pictures within each experimental trial varied in three ways according to a partial Latin Square (CAG = Wildfire, Action, Forest; GCA= Forest, Wildfire, Action; AGC = Action, Forest, Wildfire). The combinations of three orders and the four different pro-environmental behaviors resulted in a total of 12 trials being shown to each participant. In each trial, the specific pictures within each category (e.g., wildfire, action, and forest) were randomized. No picture was repeated for any participant across trials, ensuring that each picture was shown only once. Further, the order of the 12 different experimental trials was also randomized (see Figure 1 for example trial).

**Figure 1:**
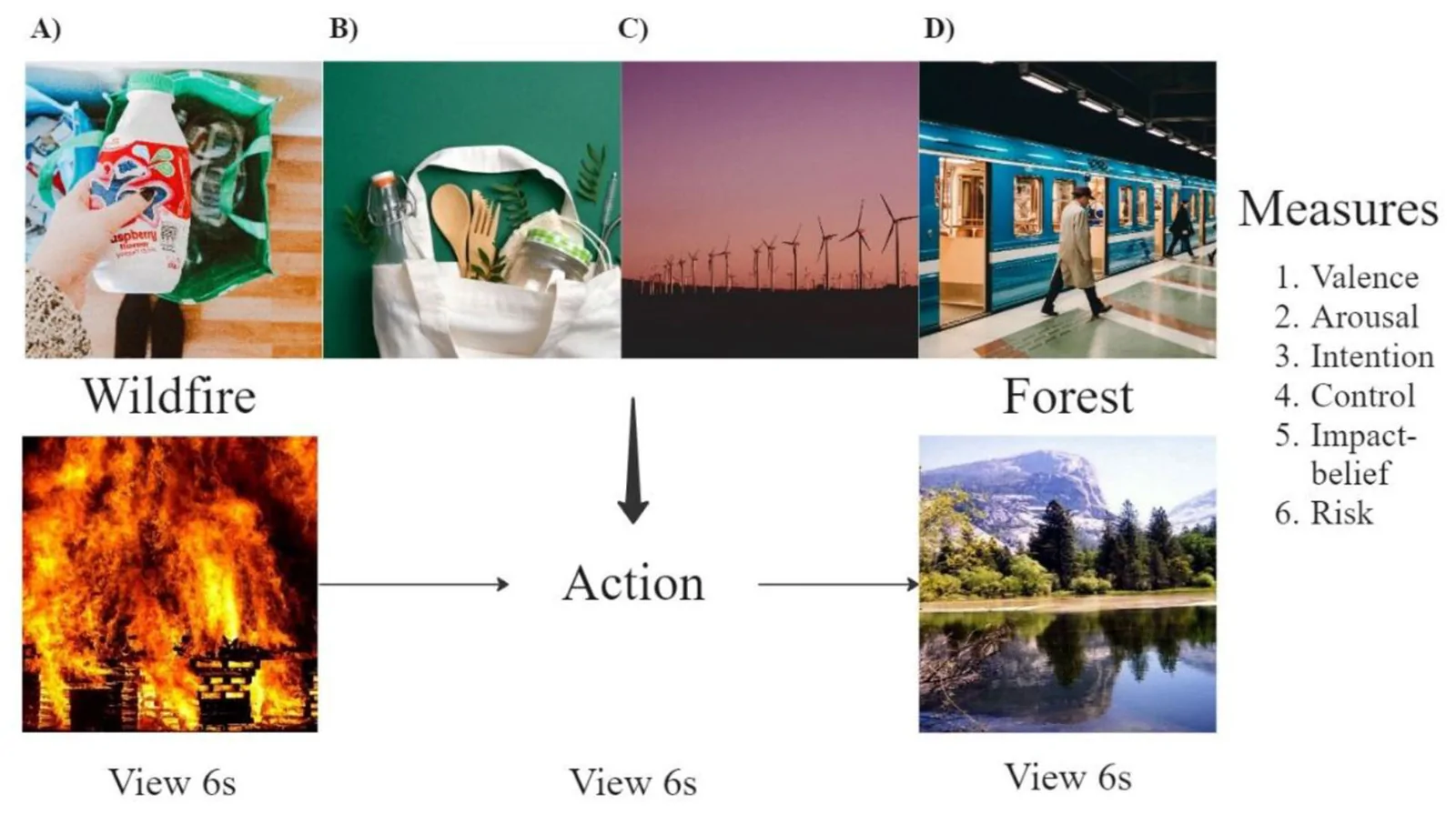
Schematic example of an experimental trial featuring wildfire, action, and forest stimulus sequence presentation. *Note*. Examples from each pro-environmental behaviors are shown, however only one action picture was shown each trial. **(A)** Recycling, **(B)** Avoid plastic,**(C)** Renewable electricity, **(D)** Shift to public transport.

After each unique trial, participants completed inter-manipulation ratings of valence, arousal, perceived risk to self, and pro-environmental intention also impact-belief for the behavior seen in the trial, using the same items as in the pre–measure. The inter–manipulation ratings also contained perceived behavioral control. Behavioral control was measured using one self-report item: “What possibility do you perceive you have to adopt this sustainable behavior?”, on a scale ranging from 0 (Not possible at all) to 100 (Completely possible).

*Picture selection*. Before conducting the experiment, we collected normative data for a total of 72 pictures (eight Avoid Plastic, eight Shift to public transport, eight renewable electricity, 8 recycling, 20 forest, 20 wildfire). The participants (*N* = 200) represented a separate American sample that were also recruited online (Prolific) and responded through an online survey tool (Qualtrics). All data were collected during a single session (22th November 2023). The pictures were sourced from various online repositories (e.g., Pixabay). For the exact origin of each picture, please refer to the Open Science Framework [OSF]. Participants viewed each of the 72 pictures for 6 s each, after each picture they rated valence and arousal, using the same measure as above. The presentation order of the pictures was randomized.

Based on the average value for each picture we selected three for each behavior to make them as comparable as possible in their level of valence across all four behaviors, so that the different action images and types would not differ systematically. However, it should be noted that all individual action images, except one, regardless of type had a positive valence (53.39–75.10). As such, images of individual pro-environmental action appear to have an inherent slightly positive valence. Further, the 12 healthy forests pictures with the most positive valence ratings, and the 12 wildfires pictures with the most negative valence ratings were selected (see Table 1 for the normative valence for the selected pictures; See OSF for normative values for all pictures). As such, environmental outcome images took two forms; negatively valenced consequences, and positively valenced goal pictures.

**Table 1:** Average and range of normative valence for selected pictures split by each picture type.

Picture type	Valence	Lowest	Highest
Avoid plastic	64.98	64.59	65.58
Recycling	64.61	63.28	65.69
Renewable electricity	66.31	66.06	66.70
Shift to public transport	64.27	60.73	67.28
Forest	79.09	76.18	83.54
Wildfire	27.37	24.62	31.02

Valence represents the average of the mean for each picture type valence ratings collected in the normative–rating study. Lowest/Highest = the lowest and highest mean valence of the pictures within that picture type.

From these, we deliberately selected the subsets that were most congruent in valence so that the four action categories (plastic reduction, recycling, transport, and renewable energy) would not differ systematically. The selected images were rated as slightly positive overall, but the key point is that they were kept consistent across categories to minimize valence-related variability in the action images themselves.

#### 2.2.2 Post-manipulation ratings

Once all trials were finished, participants provided demographic details (gender, age, state of residence) and shared their prior engagement in the four pro-environmental behaviors featured in the trials. For the shift to public transport behavior, we asked about the engagement of two transport behaviors: “How often do you travel by car?”, with five scale points ranging from 1 (Never) to 5 (Every day); “How many trips have you traveled by airplane the last 12 months?”, with four scale points ranging from 1 (0 trips) to 4 (5+ trips). To measure engagement in recycling behaviors we asked them to answer: “To what extent do you recycle your household waste?”, with four scale points ranging from 1 (None at all—I do not sort any of the household waste) to 4 (Comprehensive Sorting—I separate various types of household waste, including plastic, glass, metal, compostable waste, and more). To acquire information about their current electricity type we asked: “What source does your household electricity come from?”, with the four alternatives: Fossil fuels—For example Coal; Nuclear; Renewable—For example wind; I do not know. Lastly, to measure if they have been trying to reduce their plastic use, we asked: “To what extent have you been trying to reduce your plastic expenditure?”, with four scale points ranging from 1 (Not at all—my plastic expenditure is high) to 4 (Completely—I almost have no plastic expenditure). Just before the participants ended the survey, they received a short debriefing about the experiment. For the complete survey materials see OSF.

## 3 Results

### 3.1 Descriptive results

#### 3.1.1 Current engagement in sustainable behaviors

Most (59.27 %) participants reported that they traveled by airplane at least once a year, with the most common response being 1–2 trips per year. These results show that a majority of the participants had the potential to reduce their air travel (see Table 2). This pattern was even more apparent regarding car travel, with only 0.43 % reporting they never traveled by car, and a clear majority reported they went by car daily or most of the time (73.92 %). Regarding household electricity use, about one third of the sample (34.27%) were unaware of their electricity source. Fossil fuels were the most commonly reported source of household electricity, reported by 37.29% of participants. The most common recycling practice involved basic sorting (e.g., paper, plastic, glass, and metal). Regarding plastic use, the most frequent response was an effort to reduce consumption.

**Table 2:** Participants current engagement in sustainable behaviors.

Climate harming 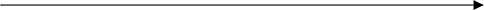 Climate change mitigation					
Air travel	5+ Trips 6.25% (29)	3–4 Trips 16.81% (78)	1–2 Trips 36.21% (168)	0 Trips 40.73% (189)	
Car travel	Every day 29.31% (136)	Most of the time 44.61% (207)	About half the time 7.97% (37)	Sometimes 17.67% (82)	Never 0.43% (2)
Electricity	Fussel fuels 37.29% (173)	Nuclear 10.13 % (47)	Renewable 18.32% (85)	Do not know 34.27% (159)	
Recycling	None 11.85% (55)	Minimal 12.72%	Basic 57.97% (269)	Comprehensive 17.46% (81)	
Plastic	High expenditure 10.99% (51)	Reduce to some extent 50.86% (236)	Reduce to high extent 34.27% (159)	Almost no expenditure 3.88% (18)	

Current engagement in pro-environmental behaviors, reported after the completed trials (*N* = 464).

### 3.2 Order and behavior effects

For the overall means of valence, arousal, control, belief, risk, and intention, along with the means split by behavior and presentation order, refer to Table 3. Linear mixed-effects models were conducted with presentation order, behavior, and their interaction as fixed effects, and participant as a random effect. Pairwise comparisons of estimated marginal means were conducted using the Holm-Bonferroni correction. The analysis revealed that the main effect of order only influenced valence, with no significant interaction effects between order and behavior. However, impact belief, behavioral control, and pro-environmental intention all demonstrated significant main effects of behavior.

**Table 3:** Overall mean, standard deviation, and confidence intervals, with means split by behavior and presentation order.

			95% CI	
Measure	Mean	SD	Low	High
Valence	66.64	22.59	66.05	67.24
Arousal	66.35	25.03	65.69	67.01
Intention	74.14	27.66	73.42	74.87
Control	69.59	29.63	68.82	70.37
Belief	72.42	26.19	71.73	73.10
Risk	64.73	30.48	63.93	65.53
	El	Plastic	Recycling	Transport
Valence	67.17	66.52	66.46	66.43
Arousal	66.87	66.17	66.28	66.09
Intention	77.39	74.39	83.88	60.91
Control	67.20	70.15	82.49	58.54
Belief	75.80	71.08	71.60	71.19
Risk	64.55	65.02	65.03	64.30
	CAG	GCA	AGC	
Valence	69.24	67.56	63.13	
Arousal	66.15	66.70	66.21	
Intention	74.10	74.44	73.90	
Control	69.85	69.81	69.12	
Belief	72.36	72.80	72.09	
Risk	64.93	64.69	64.56	

Overall means for valence, arousal, intention, perceived behavioral control and impact belief, over all trials, and split per behavior and presentation order (*N* = 464). Standard deviation (SD) and 95% confidence interval (CI) were presented for mean over all trials.

*Valence*. The main effect of presentation order on valence was significant, *F* (2, 5093) = 118.72, *p* < 0.001. *Post-hoc* pairwise comparisons revealed significant differences between all levels of order. Participants reported significantly higher valence ratings for WAF (*M* = 69.24) compared to both GCA (*M* = 67.56*, p* < 0.001), and AGC (*M* = 63.13, *p* < 0.001), as well as higher ratings after GCA compared to AGC (*p* < 0.001). These results indicate that participants felt most positive after seeing the wildfire–action–forest sequence. In contrast, the main effect of behavior on valence was not significant, *F* (3, 5093) = 1.10, *p* = 0.348, and the interaction effect between order and behavior was also not statistically significant, *F* (6, 5093) = 0.84 *p* = 0.538.

*Arousal*. When analyzing arousal, we found no significant main effect of order, *F* (2, 5093) = 1.31, *p* = 0.269, or behavior, *F* (3, 5093) = 1.36, *p* = 0.253. Additionally, there was no significant interaction effect, *F* (6, 5093) = 1.04, *p* = 0.396.

*Risk*. Similarly, we found no effect of order, *F* (2, 5093) = 1.28, *p* = 0.279, and no interaction between order and behavior type when analyzing the perceived risk of climate change, *F* (6, 5093) = 0.34, *p* = 0.917. However, we did find a significant main effect of behavior on perceived risk, *F* (3, 5093) = 3.44, *p* = 0.016. *Post-hoc* analyses indicated that participants reported lower perceived risk when the sequence included a transport picture (*M* = 64.30), in comparisons with sequences including pictures related to reducing plastic (*M* = 65.02, *p* = 0.049), and pictures of recycling (*M* = 65.03, *p* = 0.049).

*Impact Belief*. We found no effect of order, *F* (2, 5093) = 1.51, *p* = 0.221, and no interaction effect on impact-belief, *F* (6, 5093) = 0.23, *p* = 0.966. However, there was a significant main effect of behavior, *F* (3, 5093) = 45.11, *p* < 0.001. *Post-hoc* analyses indicated that participants perceived the use of renewable electricity (*M* = 75.8) to be significantly more impactful than all the other pro-environmental behaviors (*M* = 71.08–71.60, all *p* < 0.001), however no other behaviors differed from each other (all *p* > 0.05), for exact statistics see Supplementary 1.

*Control*. Similarly, when looking at behavioral control, we found no effect of order, *F* (2, 5093) = 0.80, *p* = 0.453, and no interaction effect, *F* (6, 5093) = 0.37, *p* = 0.898. However, we again found a significant main effect of behavior, *F* (3, 5093) = 353.03, *p* < 0.001. *Post-hoc* analyses indicated significant differences between all behaviors (all *p* < 0.001), with participants perceiving highest behavioral control over recycling (*M* = 82.49) and the lowest behavioral control over shifting to public transport (*M* = 58.54). For exact statistics see Supplementary 1.

*Intention*. Lastly, when exploring pro-environmental intention, we found no main effect of order, *F* (2, 5093) = 0.41, *p* = 0.666 and no interaction effect, *F* (6, 5093) = 0.10, *p* = 0.996. However, we found a significant main effect of behavior, *F* (3, 5093) = 377.64, *p* < 0.001. *Post–hoc* analyses indicated significant differences between all behavior types (all *p* < 0.001), with participant having the highest intention to recycling (*M* = 83.88) and the lowest intention to shifting to public transport (*M* = 60.91). For exact statistics see Supplementary 1.

### 3.3 Predicting intention

As a next step, we used a mixed model to examine whether valence, arousal, perceived behavioral control, impact belief, and perceived risk could predict pro-environmental intention (see Table 4). We first analyzed the role of valence and arousal as predictors of pro-environmental intention (Model 1). We then included behavioral control, impact belief, and perceived risk as predictors in the model (Model 2). Both models accounted for random intercepts for participants and behavior.

**Table 4:** Mixed model predicting intention with valence, arousal, control, belief, and risk.

Fixed effects	Model 1	Model 2
Intercept	0 [−0.35, 0.35]	0 [−0.16, 0.16]
Valence	0.09 [0.06, 0.13]^***^	0.02 [ < 0.01, 0.04]^*^
Arousal	0.10 [0.06, 0.13]^***^	−0.03 [−0.06, −0.01]^**^
Control		0.64 [0.62, 0.65]^***^
Belief		0.27 [0.24, 0.29]^***^
Risk		0.04 [ < 0.01, 0.07]^*^
Marginal *R*^2^/Conditional *R*^2^	0.028/0.556	0.661/0.793
AIC	49474.187	45155.154

Mixed Models (*N* = 464). Model 2 allowed for random intercepts for participants, and behavior. Standardized betas. ^*^p < 0.05, ^**^p < 0.01, ^***^p < 0.001.

Model 1 revealed that both valence and arousal significantly predicted pro-environmental intention. Specifically, more positive valence [β = 0.09 (0.06, 0.13), *p* = 0.031] and higher arousal [β = 0.10 (0.06, 0.13), *p* < 0.001] were associated with greater intentions to act pro-environmentally. In Model 2, all variables significantly predicted pro-environmental intention. However, the predictive value of valence [β = 0.02 (< 0.01, 0.04), *p* < 0.001] and arousal [β = −0.03 (−0.06, −0.01), *p* = 0.009] decreased from Model 1 to Model 2, suggesting that impact-belief, risk perception, and behavioral control also account for variance in affective experience.

The results from Model 2 indicate that perceiving climate change as a greater personal risk was linked to higher pro-environmental intention [β = 0.04 (< 0.01, 0.07), *p* = 0.027]. However, impact belief and perceived behavioral control were the strongest predictors. Participants who believed their actions had higher mitigation potential [β = 0.27 (0.24, 0.29), *p* < 0.001] or felt more capable of performing the behavior [β = 0.64 (0.62, 0.65), *p* < 0.001] reported a greater intention to engage in pro-environmental actions.

### 3.4 Mediation analysis

The analysis in Section 3.2 revealed that valence was significantly affected by presentation order. Additionally, the analysis in Section 3.3 indicated that valence predicts intention. While no direct effect of presentation order on intention was found, we explored the possibility of an indirect effect, where changes in valence due to presentation order influenced pro-environmental intentions. We focused the mediation analysis on valence since presentation order had no significant effect on the other measures.

We applied a Quasi–Bayesian approach to causal mediation analysis. To account for variability in pro-environmental intention that may arise from individual differences and the nature of the behavior itself, we included random intercepts for both participant and behavior in the model. The result revealed no significant direct or total effects of order on intention (See Table 5). However, the results showed a significant indirect effect—showing that less positive valence, as a result of presentation order, results in less intention act pro-environmentally.

**Table 5:** Mediation analysis—effect of presentation order on intention mediated by valence.

	Effect						
Order	Indirect/ACME	95% CI	Direct/ADE	95% CI	Total	95% CI	Proportion mediated (%)
CAG vs. AGC	−0.90^***^	[−1.19, −0.62]	0.71	[−0.59, 1.99]	−0.19	[−1.42, 1.06]	94.4
CAG vs. GCA	−0.37^***^	[−0.57, −0.20]	0.71	[−0.56, 1.90]	−0.35	[−0.83, 1.64]	39.2
GCA vs. AGC	−0.70^***^	[−0.97, −0.47]	0.14	[−1.05, 1.37]	−0.56	[−1.72, 0.65]	81.1

Quasi–Bayesian causal mediation analysis (*N* = 464). ^***^p < 0.001.

For the comparison between CAG and AGC, the indirect effect was significant [*ACME* = −0.90, 95% CI (−1.19, −0.62), *p* < 0.001], while the direct effect was non-significant [*ADE* = 0.71, 95% CI (−0.59, 1.99)]. The total effect was also non-significant [*Total Effect* = −0.19, 95% CI (−1.42, 1.06)], with 94.4% of the effect mediated.

In the comparison between CAG and GCA, the indirect effect was significant [*ACME* = −0.37, 95% CI (−0.57, −0.20), *p* < 0.001], while the direct effect was non-significant [*ADE* = 0.71, 95% CI (−0.56, 1.90)]. The total effect was non-significant [*Total Effect* = −0.35, 95% CI (−0.83, 1.64)], with 39.2% of the effect mediated.

For the comparison between GCA and AGC, the indirect effect was significant [*ACME* = −0.70, 95% CI (−0.97, −0.47), *p* < 0.001], while the direct effect was non-significant [*ADE* = 0.14, 95% CI (−1.05, 1.37)]. The total effect was non-significant [*Total Effect* = −0.56, 95% CI (−1.72, 0.65)], with 81.1% of the effect mediated.

Results indicate an indirect effect of presentation order on pro-environmental intention through valence. Specifically, less positive valence elicited by presentation order significantly decreased intention. The results further suggest that the indirect order effect on intention *via* valence were stronger for the contrast with AGC and the other two (Figure 2).

**Figure 2:**
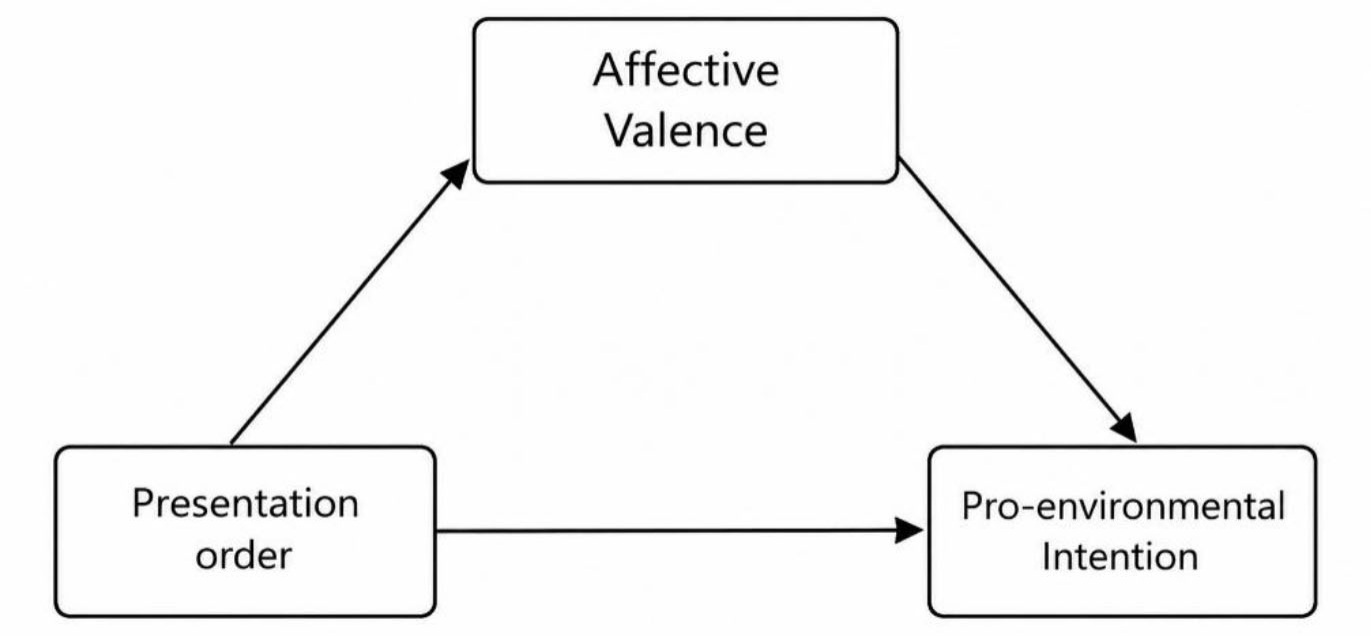
Mediation model of the effect of presentation order on pro-environmental intention through affective valence.

### 3.5 Hierarchical Bayesian computational modeling

To further investigate individuals' affective responses to climate related pictures and their relation to pro-environmental intention computational models aimed at predicting affective valence based on viewed pictures were run (see Table 6). The models that were utilized aimed to quantify specific contributions of distinct types of pictures to self–reported valence. The model was fitted to the valence data using Hierarchical Bayesian estimation, which estimates posterior distributions representing uncertainty for both group- and individual-level parameters (Gelman et al., 2014; Kruschke, 2014). The method was chosen due to its ability to balance random and fixed effects, capturing individual differences while constraining participants by group distributions. This allowed individuals to vary from the group to the extent their data is informative. We developed two models, Model 1 (Equation 1) included parameters for the picture type (Wildfire, Action, or Forest) and a parameter (**γ**) accounting for sequential placement of the picture in the presentation order (CAG, GCA, and AGC). Model 2 (Equation 2) only included parameters for picture type.


$$\begin{array}{l} Valence=w_{0}+w_{C}\cdot \gamma^{3-Order_{C}}\cdot NV_{C}+w_{A}\cdot \gamma^{3-Order_{A}}\cdot NV_{A}\\ \;+w_{G}\cdot \gamma^{3-Order_{G}}\cdot NV_{G} \end{array}$$ (1)

$$\begin{array}{ccc} Valence & = & w_{0}+w_{C}\cdot NV_{C}+w_{A}\cdot NV_{A}+w_{G}\cdot NV_{G} \end{array}$$ (2)

Here, *w*_*C*_, *w*_*A*_, and *w*_*G*_ parameters are the weights assigned to consequence, action, and goal pictures, and *w*_0_is the intercept. γ parameter (0 ≤ γ ≤ 1) is a forgetting parameter that introduces a discount for the weights depending on the presentation order in the sequence (first, second or third). Specifically, the last picture in the sequence is not discounted (γ^0^), the second picture is discounted by γ, and the first picture is discounted by γ^2^. *NV*_*C*_, *NV*_*A*_, and *NV*_*G*_ are normative valence ratings for the presented pictures collected in the normative-rating study.

**Table 6:** Mean and credible interval for the valence model 1 and 2.

Parameters	Model 1		Model 2	
(Intercept)	0.3	[0.23, 0.37]	0.22	[0.15, 0.30]
Σ	0.17	[0.16, 0.19]	0.19	[0.18, 0.20]
Goal	0.11	[0.05, 0.17]	0.07	[−0.01, 0.15]
Action	0.22	[0.12, 0.33]	0.07	[−0.04, 0.18]
Consequence	0.32	[0.22, 0.41]	−0.03	[−0.12, 0.06]
Picture placement	0.71	[0.62, 0.79]	
WAIC	−2246.78		−1738.4	

Summarizes the model parameters with the posterior distribution of the group level means and reports 95% highest density intervals (HDIs) indicating the ranges, in which the most probable 95% of values fall.

The two models were fitted to the data using Markov Chain Monte Carlo (MCMC), with 8 chains, 300 warm up iterations and 16.000 samples. The models were coded in Stan (Carpenter et al., 2017) and fitted in R using the *rstan* package (Stan Development Team, 2018). We inspected the trace plots for convergence and ensured that all *R–hat* values were under 1.01 which indicated that chains have adequate mixing (Gelman and Rubin, 1992). Initial model-fitting resulted in convergence issues. After removing participants with low response variation, same response in more than half the trials (*N* = 51), the models converged successfully. Model comparison was done according to widely applicable information criterion (WAIC), a Bayesian approach for estimating out–of–sample predictive accuracy with lower values indicating a better fit (Gelman et al., 2014; Watanabe and Opper, 2010). The results showed that the more complex Model 1, which accounts for the presentation orders, provided a better fit. Therefore, we focus on Model 1.

In addition to fitting the model, we completed posterior predicative checks (500 samples) to study how well the model performed in predicting valence (see Figure 3). The result showed that observed mean valence for each trial fell within the 95% high density interval predicted by Model 1 for all trials except trial 9. This indicates that Model 1 has good predictive power for affective valence.

**Figure 3:**
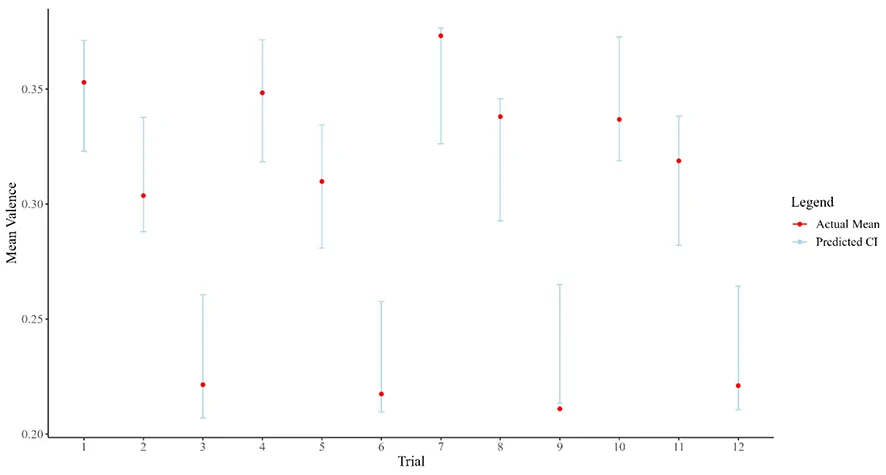
Posterior predictive checks Model 1. Posterior predictive checks for Model 1. Average valence split per trial (red point) together with 500 posterior predictive simulations from the affective experience model 1 (2.5%−97.5 % credible intervals, light blue).

When studying the parameters included in Model 1, the result showed that the model identified a strong recency effect [*posterior mean* = 0.71, 95% HDI (0.62, 0.79)]. When moving down in presentation order one step from being the last picture to being the second picture, the impact the picture on the affective experience went down by 29 %. The recency effect was further supported by a mixed model analysis (See Supplementary 2). Further, all the picture types (Wildfire, Action, and Forest) were predictive for the affective experience. In particular, the pictures of forests [*posterior mean* = 0.11, 95% HDI (0.05, 0.17)] predicted less than the action pictures [*posterior mean* = 0.22, 95% HDI (0.12, 0.33)] and with clearly separated confidence intervals from the wildfire picture [*posterior mean* = 0.32, 95% HDI (0.22, 0.41)], which in turn had the highest predictive value for valence of the three types.

Finally, we investigated Spearman rank order correlations between individual parameter estimates for the valence model and self-reported intention to study whether specific aspects of affective reactions to pictures could be related to overall pro-environmental intention (averaged over behaviors). We found that self-reported pro-environmental intention was significantly correlated with both intercept (*w*_0_; Spearman's Rho = 0.269, *p* < 0.001) and the weight parameter for action pictures (*w*_*A*_; Spearman's Rho = 0.245, *p* < 0.001). This suggests that individuals reporting overall greater pleasantness (as reflected by *w*_0_) tended to report higher pro-environmental intention. Further, those that were more heavily affected by action pictures in their valence (as reflected by *w*_*A*_) reported higher intention. The weight parameters for consequences pictures (*w*_*C*_; Spearman's Rho = −0.063, *p* = 0.246), goal pictures (*w*_*G*_; Spearman's Rho = 0.021, *p* = 0.705), and picture placement (γ; Spearman's Rho = −0.103, *p* = 0.057), did not significantly correlate with self-reported intention.

## 4 Discussion

The causal relationship between affect and pro-environmental action remains poorly understood (Brosch, 2021). Visual climate change communication typically focuses on affect-eliciting stimuli presented in isolation, instead of as a sequence experienced temporally. In the current study, three types of affect-eliciting pictures—depicting wildfires, forests, and pro-environmental behaviors—were presented sequentially, followed by measures of affective experience, pro-environmental intentions, and other antecedents of behavior. Using this design, we explored how a sequence of affective experience integrates and how that summary experience relates to pro-environmental intentions.

Consistent with previous findings, our normative rating study on climate change pictures shows that pictures depicting the consequences of climate change (e.g., wildfires) evoke negative emotional responses, while pictures representing climate change actions (e.g., recycling) lead to more positive emotional experiences, as compared to the images of consequences (Carlson et al., 2020; Chapman et al., 2016; Lehman et al., 2019). Extending these results, pictures of climate change goals (i.e., healthy forests) generated the strongest positive emotional responses. These results support the idea that climate change pictures can effectively elicit a range of affective reactions.

Moving to the main ideas assessed in the current study, we expected affective experience to vary across the different sequences, even though each sequence contained the same basic elements (i.e., wildfire, forest, and action pictures). We expected this variation to emerge both from differences in the affective valence associated with the picture types and from their placement within the sequence (i.e., recency effects). In partial support, we found a significant order effect on participants' affective valence. Participants reported feeling the most positive after viewing the wildfire-action-forest sequence, followed by the forest-wildfire-action sequence, and the least positive after viewing the action-forest-wildfire sequence. However, order did not have a significant impact on arousal. Seeking to identify the specific mechanisms underlying these effects, hierarchical Bayesian computational modeling aimed at predicting affective valence was applied. The computational model suggested that these order effects were primarily driven by differences in picture placement within the sequence. Specifically, pictures presented later in the sequence exerted a stronger influence on reported affective valence than pictures presented earlier. For each step earlier in the sequence (e.g., from third to second), predictive influence decreased by 29%. This replicates previous findings showing that people tend to give greater weight to the most recent emotional stimuli (Alaybek et al., 2022; Kahneman et al., 1993). Thus, the findings demonstrate that temporal placement within a sequence matters for affective responses to climate communication.

Additionally, picture type influenced affective responses independently of placement. Wildfire pictures had the largest impact, followed by action-oriented pictures and, lastly, goal-oriented pictures. One interpretation of these findings is that negatively valenced stimuli, such as wildfire pictures, exert a stronger influence on the integrated affective experience than positively valenced stimuli, such as goal-oriented pictures. This pattern is consistent with negativity bias accounts, suggesting that negative information receives greater weight than equally positive information during the construction of affective experience (Baumeister et al., 2001; Kesenheimer et al., 2026; Rozin and Royzman, 2001).

Additionally, picture type influenced affective responses independently of placement. Wildfire pictures had the largest impact, followed by action-oriented pictures and, lastly, goal-oriented pictures. One interpretation of these findings is that negatively valenced stimuli, such as wildfire pictures, exert a stronger influence on the integrated affective experience than positively valenced stimuli, such as goal-oriented pictures. This pattern is consistent with the broader “bad is stronger than good” framework, which proposes that negative events and information generally exert a greater psychological impact than equally positive experiences (Baumeister et al., 2001). More specifically, it aligns with negativity bias accounts suggesting that negative information receives greater weight than positive information during evaluation and decision making (Rozin and Royzman, 2001). This interpretation is consistent with recent evidence suggesting that emotions associated with environmentally harmful outcomes have a stronger influence on attitudes and behavioral decisions than emotions associated with environmentally beneficial outcomes (Kesenheimer et al., 2026).

An additional perspective is to view the sequences as a type of affective gestalt. A gestalt perspective on emotion is typically based on the notion that differing cognitive beliefs and desires come together to form a unitary emotional experience that differs from the sum of its parts (Castelfranchi and Miceli, 2009). However, we suggest that this perspective can also be applied to climate change communication stimuli, in which an affective gestalt emerges when a sequence of affect-eliciting stimuli is presented temporally. Thus, our results suggest that each sequence acquires a unique meaning, despite containing the same constituent elements.

Our findings are particularly significant because they demonstrate the possibility of developing a predictive model of valence that operates without requiring personal input data. The best-performing model included the normative valence ratings of each picture in the sequence, picture placement within the sequence, and picture type. This approach offers a promising way to forecast affective responses based on generalizable stimulus characteristics, highlighting its potential applicability across communication contexts.

Previous research has found varying effects of positive and negative emotions on intention and behavior (Brosch, 2021). Some studies suggest that increased negative affect leads to stronger pro-environmental intentions (Chapman et al., 2016), whereas others suggest that positive affect enhances these intentions (O'Neill et al., 2013). A potential reason for these diverging results may lie in the mode of evaluation—specifically the difference between joint evaluation (where distinct types of stimuli are compared by the same individual) and separate evaluation (where one type of stimulus is evaluated by an individual). Research on decision-making often shows a preference reversal between these modes, which is suggested to be driven by the availability of different qualities that become more or less salient when options are viewed in comparison to each other or in isolation (Hsee et al., 1999).

In the context of climate change pictures, studies using joint evaluation (e.g., comparing positive and negative pictures) find that positive pictures increase perceived efficacy (O'Neill et al., 2013). In contrast, studies using separate evaluations tend to show that negative pictures result in a stronger intention to change behavior (Chapman et al., 2016). This phenomenon could be viewed as a form of preference reversal or, more aptly, a *motivation reversal*—where the evaluation mode alters the emotional impact of the pictures and, consequently, the motivation to act pro-environmentally (Markowitz and Shariff, 2012). Theoretically, individuals may be driven by the desire to reduce unpleasant feelings. When affect-eliciting stimuli, such as pictures, are presented in isolation, negative affect can be alleviated by reporting intentions to act on the issue. In contrast, positive affect might lead to a diffusion of responsibility (Markowitz and Shariff, 2012), where individuals assume that others will address the problem. However, when both types of stimuli are presented jointly, a positive affect attention bias may emerge (Carlson et al., 2020), prompting individuals to engage with the positive, often action-oriented stimuli more actively.

Beyond understanding how stimuli, both separately and in combination, contribute to overall affective experience, we also explored their influence, along with other antecedents, on pro-environmental intentions. In this context, we considered pro-environmental intention as the most proximal antecedent to actual pro-environmental action (Ajzen, 1991). We found that both valence and arousal were significant predictors of intention on a trial-by-trial basis. However, the strength of their individual predictive power was reduced—though still significant—when perceived control, perceived risk, and impact-belief were added to the model. This shift suggests considerable covariation among these climate change antecedents, which future research should aim to disentangle (Clayton et al., 2015).

Combining the findings of an order effect on valence and the predictive relationship between valence and intention, we expected an indirect effect of presentation order on pro-environmental intention, mediated by valence. A mediation analysis confirmed our expectations about the indirect effect. While no direct effect of order on intention was found, there was an indirect effect *via* valence. This suggests that the variation in pleasantness caused by the different stimulus orders influenced participants' intentions to act pro-environmentally. These results suggest that manipulating affective experiences may influence pro-environmental intentions—in this study, more positive affective valence was associated with higher intention. This study provides initial evidence of a causal relationship between affective valence and pro-environmental intention (Brosch, 2021). The finding motivates the need for research examining the causality between affect, intentions and subsequent pro-environmental behaviors (Brosch and Sauter, 2023; Schneider and van der Linden, 2023).

In addition, we correlated participants' parameter weightings from the predictive model with their reported levels of intention. This analysis revealed that individuals who, on average, felt more positive during the trials also reported higher intentions. Additionally, the results showed that participants who were more influenced by the action pictures reported higher intention. This makes intuitive sense, as those who place more weight on their emotional response to action pictures are more likely to want to act in line with those pictures. Suggesting that using action pictures might be particularly effective to motivate pro-environmental action in subgroups of the population. Further, the pictures might work as a type of visual *implementation intention* manipulation (Armitage, 2004).

Lastly, stimuli such as those used here, could serve as a means to manipulate integral core affect to test proposed emotion driven reinforcement processes (Baumeister et al., 2007; Brosch, 2021; Schneider et al., 2021; Schneider and van der Linden, 2023). For instance, one could utilize wildfire pictures to evoke negative integral affect related to air travel. Negative affect could foster an avoidance motivation aimed at promoting climate change mitigation behaviors. Conversely, forest pictures could be employed to generate positive integral affect associated with alternative modes of transportation, such as trains and buses. Positive affect could then in turn inspire an approach motivation toward these mitigation friendly travel options.

### 4.1 Limitations

To the authors' knowledge, this is the first study to explore the effect of affective integration in climate change communication research. However, the generalizability of the findings should be interpreted with caution. Notably, the study only examined four pro-environmental behaviors. While no unique affective response specific to behavior was found, covariation with other antecedents could indirectly influence affect. Additionally, the action pictures varied in important ways, particularly in their level of abstraction. For example, 'reducing plastic' was difficult to depict directly, so the authors represented it indirectly using alternative materials (e.g., fabric bags or paper straws).

Throughout the paper, we distinguish between three types of climate change pictures: action (as noted above), consequence, and goal. However, the latter two categories were only represented by wildfires and healthy forests, respectively. Future research should aim to replicate these findings using pictures of other climate-related risks, such as flooding and drought. Similarly, future studies should evaluate the generalizability of the goal picture category, by including a broader range of positive environmental outcomes and healthy natural environments.

Further, the experiment did not employ a fully orthogonal design. While the consequence images included both positively and negatively valenced content, the pro-environmental action images were consistently slightly positive in valence. This design allowed us to explore the inherent affective tone typically associated with action imagery, enhancing ecological validity and facilitating transferability to climate change communication. Consequently, we cannot determine whether the observed differences were driven by image type, emotional valence, or a combination of the two. Future studies should address this limitation by orthogonalizing image type and valence, for example by norming a broader range of climate-related images to identify both positively and negatively valenced examples within each image category, or by manipulating valence through affective conditioning, such as pairing images with emotionally evocative sounds. This would allow for a clearer interpretation of whether observed effects are driven by image type, emotional valence, or their interaction.

Another factor that may influence affective and behavioral responses is the coherence between images in a sequence. Clear causal storytelling, such as renewable energy reducing wildfire risk and leading to a healthy forest, may be more persuasive than less coherent sequences (Jones and Peterson, n.d.). Although not examined in this study, future research should explore whether such coherence moderates emotional responses and pro-environmental intentions.

A limitation of the study is the relatively small number of trials (12), which may constrain the reliability of trial-specific ratings. However, the emergence of theoretically expected patterns, such as a recency effect and an effect of order on valence ratings, as well as successful prediction of affective responses by the computational model, suggests that the affective data captured meaningful variation and were adequate for testing our hypotheses.

A key limitation of this study is the absence of an objective behavioral measure. Environmental psychology has been criticized for its lack of empirical evidence on actual behavior (Nielsen et al., 2024). Future studies should aim to assess the complete chain of antecedents leading to behavior. However, as an initial study, we view this as proof of concept.

Finally, an important limitation relates to the duration participants viewed each picture. A proportion of participants had to be excluded due to this issue, potentially skewing the results. Nonetheless, we believe this is unlikely to have significantly impacted on the findings, as the viewing time issues were likely to be due to internet connection problems rather than factors relevant to the concepts being evaluated.

### 4.2 Conclusion

Affect should be considered when communicating climate change-related issues to the public. In this study, we examined how combining climate change pictures into sequences influenced affective experience. The results showed that affective valence was influenced by the order in which pictures were presented. Furthermore, hierarchical Bayesian computational modeling indicated that affective experience was neither a simple sum nor an average of participants' responses to individual stimuli. Instead, participants placed greater weight on the final picture in each sequence and were more strongly influenced by negatively valenced wildfire pictures than by positively valenced forest pictures. Together, these findings suggest that affective integration in climate change communication is shaped by both recency effects and the stronger weighting of negatively valenced climate-related stimuli. Furthermore, presentation order indirectly influenced pro-environmental intentions through changes in affective valence. Participants who reported more positive affective experiences overall, and those whose affective responses were more strongly influenced by action pictures, also reported stronger pro-environmental intentions. These findings highlight the importance of considering affective integration when designing climate change communication and suggest that computational models based on stimulus characteristics may offer a useful way of predicting affective responses without requiring personal input data.

## Data Availability

The datasets presented in this study can be found in online repositories. The names of the repository/repositories and accession number(s) can be found below: https://osf.io/mr2f4/?view_only=f16a810cd4a44e2cbc8d4c8a7cf412c5.
