# Supplementary material for: Affective integration in climate change communication: recency effects and negativity bias in responses to climate picture sequences
Source: Front Psychol. 2026 Sep 21;17:1797253. doi: 10.3389/fpsyg.2026.1797253 (PMC13634848; doi:10.3389/fpsyg.2026.1797253)
Supplement: Supplementary file 1 [file Table_1.docx]

# Supplementary materials –

# **Affective Integration in Climate Change Communication: The Role of Picture Sequences in Shaping Pro-Environmental Intentions**

Contents

[Supplementary 1 – Bonferroni-Holm Post-hoc	2](#_Toc180392357)

[Impact-Belief	2](#_Toc180392358)

[Perceived Behavioral Control	3](#_Toc180392359)

[Pro-environmental Intention	4](#_Toc180392360)

[Supplementary 2 –	5](#_Toc180392361)

[Recency effect	5](#_Toc180392362)

## Supplementary 1 – Bonferroni-Holm Post-hoc

### Impact-Belief

**Post-hoc tests with Bonferroni-Holm adjustment**

| **Contrast** | **Estimate** | **SE** | **z** | **p (Holm)** |
| --- | --- | --- | --- | --- |
| Electricity – Plastic | 4.72 | 0.48 | 9.89 | < .001 |
| Electricity – Recycling | 4.20 | 0.48 | 8.80 | < .001 |
| Electricity – Transport | 4.62 | 0.48 | 9.67 | < .001 |
| Plastic – Recycling | -0.52 | 0.48 | -1.08 | .838 |
| Plastic – Transport | -0.10 | 0.48 | -0.22 | .838 |
| Recycling – Transport | 0.41 | 0.48 | 0.86 | .838 |

**Note.** Pairwise comparisons of estimated marginal means averaged across presentation order. p values were adjusted using the Holm correction.

### Perceived Behavioral Control

**Post-hoc tests with Bonferroni-Holm adjustment**

| **Contrast** | **Estimate** | **SE** | **z** | **p (Holm)** |
| --- | --- | --- | --- | --- |
| Electricity – Plastic | -2.95 | 0.75 | -3.95 | < .001 |
| Electricity – Recycling | -15.29 | 0.75 | -20.50 | < .001 |
| Electricity – Transport | 8.66 | 0.75 | 11.62 | < .001 |
| Plastic – Recycling | -12.35 | 0.75 | -16.55 | < .001 |
| Plastic – Transport | 11.61 | 0.75 | 15.57 | < .001 |
| Recycling – Transport | 23.96 | 0.75 | 32.11 | < .001 |

**Note.** Pairwise comparisons of estimated marginal means averaged across presentation order. p values were adjusted using the Holm correction.

### Pro-environmental Intention

**Post-hoc tests with Bonferroni-Holm adjustment**

| **Contrast** | **Estimate** | **SE** | **z** | **p (Holm)** |
| --- | --- | --- | --- | --- |
| Electricity – Plastic | 3.00 | 0.70 | 4.26 | < .001 |
| Electricity – Recycling | -6.49 | 0.70 | -9.23 | < .001 |
| Electricity – Transport | 16.48 | 0.70 | 23.41 | < .001 |
| Plastic – Recycling | -9.49 | 0.70 | -13.49 | < .001 |
| Plastic – Transport | 13.48 | 0.70 | 19.15 | < .001 |
| Recycling – Transport | 22.97 | 0.70 | 32.64 | < .001 |

**Note.** Pairwise comparisons of estimated marginal means averaged across presentation order. p values were adjusted using the Holm correction.

### Perceived risk

**Post-hoc tests with Bonferroni-Holm adjustment**

| **Contrast** | **Estimate** | **SE** | **z** | **p (Holm)** |
| --- | --- | --- | --- | --- |
| Electricity – Plastic | -0.47 | 0.28 | -1.71 | .330 |
| Electricity – Recycling | -0.48 | 0.28 | -1.74 | .330 |
| Electricity – Transport | 0.25 | 0.28 | 0.91 | .726 |
| Plastic – Recycling | -0.01 | 0.28 | -0.03 | .979 |
| Plastic – Transport | 0.72 | 0.28 | 2.62 | .049 |
| Recycling – Transport | 0.73 | 0.28 | 2.65 | .049 |

**Note.** Pairwise comparisons of estimated marginal means averaged across presentation order. p values were adjusted using the Holm correction.

## Supplementary 2 –

## Recency effect

Formula: AE_t ~ 1 + Past_AE + image1 + image2 + image3 + (1 | Participant)

REML criterion at convergence: 1829.8

**Random effects**

| Groups | Name | Variance | Std.Dev. |
| --- | --- | --- | --- |
| Participant | (Intercept) | 0.07525 | 0.2743 |
| Residual |  | 0.06744 | 0.2597 |

Note. Number of obs: 4956, groups: Participant, 413, same participants as the hierarchal Bayesian model

**Fixed effects**

| Predictor | Estimate | Std. Error | df | t value | Pr(>\|t\|) |
| --- | --- | --- | --- | --- | --- |
| (Intercept) | 0.1986 | 0.0268 | 3045 | 7.420 | 1.51e-13 *** |
| Past_AE | 0.2002 | 0.0131 | 4921 | 15.302 | < 2e-16 *** |
| image1 | 0.0367 | 0.0524 | 4436 | 0.700 | 0.4839 |
| image2 | 0.0367 | 0.0523 | 4436 | 0.701 | 0.4831 |
| image3 | 0.1686 | 0.0523 | 4436 | 3.226 | 0.00126 ** |

Note. Signif. codes: 0 ‘***’ 0.001 ‘**’ 0.01 ‘*’ 0.05 ‘.’ 0.1 ‘ ’ 1
